# Mental Health, Mental Health Care, and Mental Health Reforms in Hungary in the Central/Eastern European Context: Progress, Parallels, and Persistent Gaps

**DOI:** 10.1192/j.eurpsy.2026.10758

**Published:** 2026-07-31

**Authors:** R. Wernigg, D. Őri, T. Bulyáki, A. I. Slezák, J. Harangozó, I. Gallai, Z. Guerrero, A. Kagstrom, P. Winkler

**Affiliations:** 1 Department for Primary Care Planning and Development, National Directorate-General for Hospitals; 2 Awakenings Foundation; 3 President, Hungarian Psychiatric Association; 4 Institute of Behavioural Sciences, Semmelweis University; 5 Department of Mental Health, Heim Pal National Institute of Pediatrics; 6 Department of Social Work, Eötvös Lóránt University Faculty of Social Sciences; 7 Community Mental Health Center, Zugló Healthcare Services; 8 Expert by experience, Awakenings Foundation, Budapest, Hungary; 9 Department of Public Mental Health, National Institute of Mental Health, Klecany; 10 Collaborating Centre for Public Mental Health Research and Service Development, WHO, Prague, Czech Republic; 11 Department of Global Public Health, Karolinska Institutet, Stockholm, Sweden; 12 Collaborating Center for Public Mental Health Research and Service Development, WHO, Prague, Czech Republic; 13 Scientific Lead and Team Coordinator, European Alliance Against Depression, Frankfurt am Main, Germany

## Abstract

**Introduction:**

Central and Eastern European (CEE) mental health systems share a legacy of hospital-centrism, fragmented governance, and low investment (~3.2% of health spending, largely to psychiatric hospitals). COVID-19-era telepsychiatry, nongovernmental organization (NGO) innovations, and war-related pressures catalysed activity, however, implementation and evaluation remain uneven.

**Objectives:**

To position Hungary’s mental healthcare within a unified CEE framework using a multi-country scoping review aligned with the WHO Mental Health Care Pyramid.

**Methods:**

Narrative policy/systems review (2017–2024) of national statistics and reports, OECD/WHO documents, programme evaluations, workforce registers, and inputs from professional bodies and NGOs.

**Results:**

Economic burden of mental health was ~3.1% of GDP (2018). Unmet need for mental healthcare rose from 3.8% (2021) to 8.4% (2024). Suicide declined from 46.1/100,000 (1984) to 15.7 (2019) but increased to 16.8 (2024), with persistently higher male mortality. Governance is highly centralised (Ministry of Interior/OKFŐ). Recent reforms include large salary increases (physicians ~120%, nurses ~70–80%), nationwide e-health records, and EU-funded infrastructure upgrades. Care is free at the point of use; the state covers ~87.4% of psychotropic costs. Workforce shortages mirror CEE patterns: only ~4,201 of ~8,200 registered mental-health professionals are active, with pronounced urban–rural inequities; child and adolescent services remain under-resourced. Hospital-orientation persists in Hungary. Telepsychiatry expanded during COVID-19, however, the availability, and the intra-sectoral and cross-sectoral integration of community services is widely variable. Complex NGO-led initiatives show promise yet lack national embedding. Prevention and promotion (e.g., iFightDepression; school- and work-based activities; primary-care involvement; mental-health promotion centres; labor market developments), are promising yet fragmented and insufficiently prioritized. Perinatal care assets—health-visitor network and a specialised maternity mental-health unit—are under-leveraged. Several reforms remain pilots, sustainability plans are limited, and performance monitoring favours administrative over patient-centred outcomes, echoing regional gaps.

**Conclusions:**

Hungary’s trajectory partially typifies the CEE region: key enablers (central governance, salary reform, digitalisation, infrastructure, employment) have advanced; however, hospital-centrism, workforce constraints, and cross-sector fragmentation persist. Priorities are a comprehensive national mental-health strategy; sustained, prevention-oriented financing for community services; systematic health–social–education integration; transparent, outcome-focused monitoring and evaluation; and meaningful service-user involvement to improve quality and equity.

**Disclosure of Interest:**

R. Wernigg Employee of: a back-office institute of the Hungarian Ministry of Interior., D. Őri: None Declared, T. Bulyáki: None Declared, A. Slezák: None Declared, J. Harangozó: None Declared, I. Gallai: None Declared, Z. Guerrero: None Declared, A. Kagstrom: None Declared, P. Winkler: None Declared

